# 

**Published:** 1878-08

**Authors:** 


### Chicago Medical Journal

AND

### EXAMINER.

Vol. XXXVII.—AUGUST, 1878.—No. 2.

### To our Readers and Correspondents.

Beginning with Vol. XXXVII., July, 1878, the Chicago Medical Journal and Examiner prints all records of length and weight in terms of the Metric System, and all records of temperature in degrees of the Centigrade Scale. The Metric System, legalized in the United States and Great Britain, is extensively or exclusively employed by other civilized nations, and has thus become an essential part of the international language of science. It is recommended for adoption to the profession in this country by the American Medical Association and other scientific bodies. To-day no physician can afford to be ignorant of its value, its simplicity and the meaning of its terms.

The subjoined tables and scales, which have been kindly prepared for us by Prof. W. S. Haines, will be continuously reproduced in subsequent numbers of this journal, for the ready reference of our readers and correspondents.

### Metric Measures of Length.

| | | | |
|---|---|---|---|
| Millimeter | 0.001 of a Meter | 0.03937 inches. | |
| Centimeter | 0.01 " " | 0.39370 | " |
| Decimeter | 0.1 " " | 3.93707 | " |
| **Meter** | **1. Meter** | **39.37079** | " |
| Decameter | 10. Meters | 393.70790 | " |
| Hectometer | 100. " | 3937.07900 | " |
| Kilometer | 1000. " | 39370.79000 | " |

### Metrical Weights.

| | | | |
|---|---|---|---|
| Milligram | 0.001 of a Gram | 0.015 grains. | |
| Centigram | 0.01 " " | 0.154 | " |
| Decigram | 0.1 " " | 1.543 | " |
| **Gram** | **1. Gram** | **15.432** | " |
| Decagram | 10. Grams | 154.323 | " |
| Hectogram | 100. " | 1543.234 | " |
| Kilogram | 1000. " | 15434.348 | " |

☞ The United States "nickel" five cent piece weighs five grams, and is two centimeters in diameter.

### Approximate Equivalent of Metrical Weights.

*For Rapid Reference.*

| *Milligrams.* | *Grains.* |
|---|---|
| 1 (written 0.001 or \|001)* | $\frac{1}{65}$ |
| 2 | $\frac{1}{32}$ |
| 3 | $\frac{1}{22}$ |
| 4 | $\frac{1}{16}$ |
| 5 | $\frac{1}{13}$ |
| 6 | $\frac{1}{11}$ |
| 7 | $\frac{1}{9}$ |
| 8 | $\frac{1}{8}$ |
| 9 | $\frac{1}{7}$ |

| *Decigrams.* | *Grains.* |
|---|---|
| 1 (written 0.1 or \|1) | $1\frac{1}{2}$ |
| 2 | 3 |
| 3 | $4\frac{1}{2}$ |
| 4 | 6 |
| 5 | $7\frac{1}{2}$ |
| 6 | 9 |
| 7 | 11 |
| 8 | $12\frac{1}{2}$ |
| 9 | 14 |

| *Centigrams.* | *Grains.* |
|---|---|
| 1 (written 0.01 or \|01) | $\frac{1}{6}$ |
| 2 | $\frac{1}{3}$ |
| 3 | $\frac{6}{13}$ |
| 4 | $\frac{7}{11}$ |
| 5 | $\frac{3}{4}$ |
| 6 | $\frac{9}{10}$ |
| 7 | 1 |
| 8 | $1\frac{1}{4}$ |
| 9 | $1\frac{1}{3}$ |

| *Grams.* | *Grains.* |
|---|---|
| 1 (written 1. or \|1) | 15 |
| 2 | 30 |
| 3 | 46 |
| 4 | 61 |
| 5 | 77 |
| 6 | 92 |
| 7 | 108 |
| 8 | 123 |
| 9 | 139 |

A Kilogram=$2\frac{1}{5}$ lbs. Avoirdupois.

* The decimal *line* instead of points makes errors impossible.

### Metric Fluid Measures.

In prescription writing, fluids are usually measured in Cubic Centimeters, that is, a volume represented by a cube all of whose sides measure one centimeter. An ordinary back-gammon die is usually about this size. One cubic centimeter (written 1 C. C.) = 16.231 minims. It is approximately regarded as one fourth of a fluid drachm.

### Approximate Equivalents of Cubic Centimeter.

| | | | | |
|---|---|---|---|---|
| 0.001 | C. C. | = ......... | $^1/_{65}$ | minim. |
| 0.01 | " | = ......... | $^1/_6$ | " |
| 0.1 | " | = ......... | $1^1/_2$ | " |
| **1.** | **C. C.** | **= .................** | **15** | **minim.** |
| 4. | " | = ......... | 1 | fluid drachm. |
| 16. | " | = ......... | 4 | fluid drachms. |
| 32. | " | = ......... | 1 | ounce. |

1000 C. C. (usually known as a **Liter**) is a trifle more than one quart, wine measure.

The following prescription—

℞: Potassii bromidi, ℥i.
Elixir aurantii, fl., ℥viij.
M.

Would, in metric terms, be written:

Potassic bromide, 32 Gm.

Orange elixir, 250 C. C.

Mix.

Or, in more finished decimal manner:

Potassic bromide, 30 Gm.

Orange elixir, 250 C. C.

Mix.

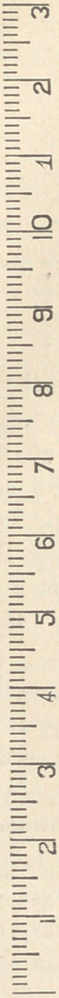

CENTIMETERS AND MILLIMETERS.

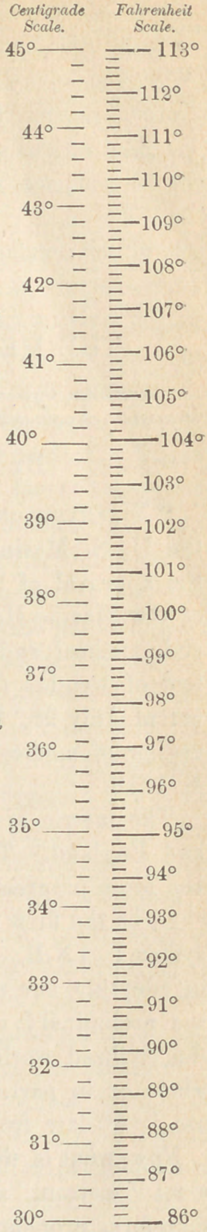

### BOOKS AND PAMPHLETS RECEIVED.

House Drainage and Water Service in Cities, Villages and Rural Neighborhoods; with Incidental Considerations of Causes Affecting the Healthiness of Dwellings. By J. C. Bayles, Editor of "*The Iron Age*," etc. Cloth, pp. 360. New York: Published by David Williams, 83 Reade street. 1878.

Lessons in Laryngoscopy; Including Rhinoscopy and the Diseases of the Throat. By Prosser James, M. D., M. R. C. P., Lecturer on Materia Medica and Therapeutics at the London Hospital; Physician to the Hospital for the Diseases of the Throat, etc., etc. Second edition, with colored plates. London: Balliere, Tindall & Cox, 20 King William street. Cloth, pp. 176. 1878.

Antagonism of Alcohol and Diphtheria. By E. W. Chapman, A. M., M. D., formerly Professor of Materia Medica and Therapeutics, etc., etc. Cloth, pp. 98. Union Argus Steam Printing Establishment. Brooklyn. 1878.

Ninth Annual Report of the State Board of Health of Massachusetts. January, 1878. Cloth, pp. 529. Boston; Rand, Avery & Co., printers to the Commonwealth, 117 Franklin street.

Practical Chemistry for Medical Students. By M. M Pattison Muir, F. R. S. E., Prælector in Chemistry, Gonville and Gains College, Cambridge. London: Macmillan & Co., 1878; pp. 64, cloth. 60c.

Lectures on Clinical Medicine; Delivered in the Royal and Western Infirmaries of Glasgow. By Dr. McCall Anderson, Professor of Clinical Medicine in the University of Glasgow. With illustrations. London: Macmillan & Co., 1878. Cloth, pp. 264. $3.00.

Insanity in Ancient and Modern Life; with Chapters on its Prevention, By Daniel Hache Tulne, M. D., Fellow of the Royal College of Physicians. London. London: Macmillan & Co., 1878. Cloth, pp. 226. $1.75.

A Practical Treatise on Aural Surgery. By H. Macnaughton Jones, M. D., M. Ch., F. R. C. S. I. and Edin. Philadelphia: Lindsay & Blakiston, 1878. Cloth, pp. 172.

Manual of Operative Surgery. By Lewis A. Stinson, B. A., (Yale) M. D. With 332 illustrations. Philadelphia: Henry C. Lea, 1878. Cloth, pp. 477. $2.50.

Nervous Diseases, their Description and Treatment. By Allan M'Lane Hamilton, M. D., Fellow of the New York Academy of Medicine, etc., etc. With fifty-three illustrations. Philadelphia: H. C. Lea, 1878.

The Atlantic Islands as Resorts of Health and Pleasure. By G. S. W. Benjamin, Author of "Contemporary Art in Europe," etc., etc., etc. Illustrated. New York: Harper Brothers, 1878. Cloth, pp. 274, $3.00. Jansen, McClurg & Co., Chicago.

Visions; a Study of False Sight. By Ed. H. Clarke, M. D.; with an introduction and memorial sketch by Oliver Wendell Holmes, M. D. Houghton, Osgood & Co., Boston, 1878. Chicago: Jansen, McClurg & Co. Cloth, pp. 315 $1.50.

Towne's Manual of Chemistry, Theoretical and Practical. Revised and Corrected by Henry Watts, B. A., F. R. S. A new American from the twelfth English edition. Edited by Robert Bridges, M. D., Professor of Chemistry in the Philadelphia College of Pharmacy; 177 illustrations. 1878. Philadelphia: H. C. Lea. Chicago: Jansen, McClurg & Co. Cloth, pp. 1027. $2.50.

Transactions of the American Gynæcological Society—Vol. II, 1877. Cloth, pp. 697. $6.50. Houghton, Osgood & Co., Boston. Jansen, McClurg & Co., Chicago.

Proceedings of Chicago Society of Physicians and Surgeons, 1874. Presented by Chicago Society of Physicians and Surgeons.

Sanitary and Medical Reports for 1873–4, by the Officers of the U. S. Navy. Prepared for publication under the direction of the Surgeon of the Navy. By H. C. Nelson, M. D., Surgeon to the U. S. Navy. Washington: Government Printing Office: 1875. Pp. 818. Presented by Chicago Society of Physicians and Surgeons.

Catalogue of the Library of the Surgeon General's Office, U. S. Army. 3 vols. Washington: Government Printing Office; 1874. Presented by Chicago Society of Physicians & Surgeons.

Cyclopædia of the Practice of Medicine. Ziemssen. Vol. XIII. Diseases of the Nervous System. New York: Wm. Wood & Co., 28 Great Jones street. Chicago: W. T. Keener, 94 Washington street.

Do Phosphureto de Tinco Sua Acçao Physiologico et Therapeutica. By Dr. D. A. Martins Costa. Rio de Janeiro, 1877.

Zur Behandlung der Blutungen nach Abort. Von Paul F. Mundé in New York.

Treatment of Chronic Aural Discharges. By Julian J. Chisholm, M. D. Read before the Baltimore Academy of Medicine. Revised from *North Carolina Medical Journal*, May, 1878.

On the Necessity of Caution in the Use of Chloroform During Labor. By W. T. Lush, M. D. New York.

Report on Monomania. By D. A. Morse, M. D., London, Ohio, 1874.

A Case of Vaginal Ovariotomy. By William Goodell, A. M., M. D., Philadelphia. Reprint from Vol. II, Gynæcological Transactions, 1878.

Case of J. W Finley, with Autopsy of the Brain. Read before the Iowa

State Medical Society, Iowa, Jan. 30, 1878. By Walter Hay, M. D., Dubuque, Iowa.

Amputations and Excisions of the Cervix Uteri; Their Indications and Methods. By J. Byrne, M. D., M. R. C. S. E., etc., etc. Reprint from Vol. II, Gynæcological Transactions, 1878.

Relative to the Work of Health Officers and of Local Boards of Health in Michigan. Circular No. 28, from the State Board of Health of Michigan

Relative to Notices of Diseases which Endanger the Public Health; Duties of Householders, Physicians and Others. Circular to Supervisors and other Officers of Townships, from the State Board of Health of Michigan.

Atlas of Skin Diseases. By Louis A. Duhring, M. D., Professor of Skin Diseases in the Hospital of the University of Pennsylvania, etc. Part III. Philadelphia: J. B. Lippincott & Co. Chicago: Jansen, McClurg & Co.

Du Petit Lait et du Lait dans la Phthisie Pulmonaire. Par Charles Paul Simon. Presented by Chicago Society of Physicians and Surgeons.

Einiges über Apoplexie. Presented by Chicago Society of Physicians and Surgeons.

Certain Symptoms of Nervous Exhaustion. By George M. Beard, Fellow of the New York Academy of Medicine, etc., etc. Read before the New York Academy of Medicine, April 4, 1878. Reprint from *Virginia Medical Monthly*, June, 1878.

The Illinois State Medical Register, 1878–9. Published annually under the supervision of the Chicago Medico-Historical Society, with the co-operation of the Illinois State Medical Society. Chicago List. Editor, D. W. Graham, A. M., M. D. Chicago: W. T. Keener, 94 Washington st.

Neuralgia and its Modern Therapeutics. By James A. Baird, M. D., Atlanta. Reprint from the Transactions of the Medical Association of Georgia.

The Functions of the Anal Sphincters, So-called. By James R. Chadwick M. D., Boston. Reprint from Vol. II, Gynæcological Transactions, 1877.

Cases of Double Uterus and Vagina. By James R. Chadwick, M. D. Read before the Obstetrical Society of Boston.

Transactions of the South Carolina Medical Association. Twenty-seventh Annual Session, held in Charleston, S. C., April 10 and 11, 1877. Charleston, S. C.: Edward Perry, Printer. Presented by Dr. Manning Simons.

Higher Medical Education the True Interest of the Public and of the Profession. An address introductory to the 112th course of lectures in the Medical Department of the University of Pennsylvania, delivered Oct. 1, 1877, by William Pepper, A. M., M. D, Professor of Clinical Medicine. Published by order of the Board of Trustees and at the request of the medical class.

### ANNOUNCEMENTS FOR THE MONTH.

### SOCIETY MEETINGS.

Chicago Medical Society—Mondays, Aug. 5 and 19.
West Chicago Medical Society—Mondays, Aug. 12 and26.

### CLINICS.

MONDAY.

Eye and Ear Infirmary—2 to 3 p. m., Ophthalmological, by Prof. Holmes; 3 to 4 p. m., Otological, by Prof. Jones.
Mercy Hospital—2 to 3 p. m., Surgical, by Prof. Andrews.
Rush Medical College—2:30 p. m., Dermatological and Venereal, by Dr. Hyde.

TUESDAY.

Mercy Hospital—2 p. m., Medical, by Prof. Hollister.

WEDNESDAY.

Mercy Hospital—2 p. m., Eye and Ear, by Prof. Jones.
Rush Medical College—4 p. m., Diseases of the Chest, by Dr. E. Fletcher Ingals.

THURSDAY.

Mercy Hospital—2 p. m., Medical, by Prof. Davis.
Rush Medical College—1:30 p. m., Neurological, by Prof. Lyman.
Eye and Ear Infirmary—2 to 3 p. m., Ophthalmological, by Dr. Hotz.

FRIDAY.

Mercy Hospital—2 p. m., Medical, by Prof. Davis.

SATURDAY.

Rush Medical College—2 p. m., Surgical, Prof. Gunn.
Chicago Medical College—2 p. m., Surgical, by Profs. Andrews and Isham; 3 p. m., Diseases of the Chest, by Prof. Johnson.

Special Clinics daily, from 2 to 4 p. m., at the South Side Dispensary, and at the Central Free Dispensary.

